# Selective nuclear elimination in multinucleate cells

**DOI:** 10.18632/oncotarget.5450

**Published:** 2015-09-01

**Authors:** Daisuke Maruyama, Tomokazu Kawashima, Tetsuya Higashiyama

**Affiliations:** Division of Biological Science, Graduate School of Science, Institute of Transformative Bio-Molecules (ITbM), and Institute for Advanced Research, Nagoya University, Chikusa-ku, Nagoya, Aichi, Japan

**Keywords:** Chromosome Section, cell fusion, multinucleate cell, nuclear elimination, programmed cell death

A few hours of fumigation will easily eliminate a home's pest infestation. However, one has to choose other means of pest control if the home's residents cannot relocate. The same issue underpins selective nuclear elimination in multinucleate cells. Recently, we reported a novel cell elimination strategy in early developing seeds of the model plant *Arabidopsis thaliana*; a large embryo-nourishing cell termed the endosperm absorbs the synergid cell [1]. The synergid serves as a beacon for the pollen tube, which conveys the sperm cells, by secreting attractant peptides. After successful fertilization, cell-fusion and subsequent nuclear disorganization rapidly inactivates the synergid, terminating pollen tube attraction and thereby preventing multiple fertilizations of the egg cell. To our surprise, this nuclear elimination is restricted specifically to the synergid-derived nucleus, even though the endosperm-derived nuclei share the same cytoplasm. The endosperm would not produce destructive molecules such as proteases or nucleases because these enzymes would also harm their own nuclei. How then does the endosperm selectively eliminate the synergid-derived nucleus after the synergid-endosperm fusion?

In *Arabidopsis thaliana*, degeneration of the synergid nucleus can be induced by 1-aminocyclopropane-1-carboxylic acid (AAC) treatment even before fertilization. AAC is a precursor of ethylene, a gaseous hormone regulating various developmental processes including fruit ripening and senescence. Ethylene signaling mutants are defective in synergid nuclear degeneration and frequently attract second pollen tubes [2]. However, ethylene signaling is activated not only in the synergid, but also in the zygote and endosperm nuclei soon after fertilization. Furthermore, the cytoplasm of the synergid and endosperm become rapidly mixed through active protoplasmic streaming. Therefore, selective elimination of the synergid nucleus through ethylene signaling should be initiated before the synergid-endosperm fusion although the detailed mechanism is still unclear. After the fusion, the synergid chromosomes appear to segregate, although fail, during the endosperm mitosis. An M-phase marker indicated that the cell cycle of the synergid nucleus is not synchronized with that of the endosperm nuclei, likely causing premature mitotic entry of the synergid nucleus propelled by the actively proliferating endosperm. Another factor controlling the mitosis-associated nuclear elimination is the endosperm-specific Polycomb Repressive Complex 2, FIS-PRC2 [1, 3]. In FIS-PRC2 mutants, the synergid nucleus persists even after several rounds of endosperm proliferation. PRC2 induces gene silencing via tri-methylation of H3K27, suggesting a role for factors downstream of FIS-PRC2. The current model is that 1) after fertilization, the synergid nucleus receives an ethylene signal and becomes susceptible to nuclear elimination; 2) the synergid and endosperm cells fuse via an unknown mechanism; 3) factors controlled by FIS-PRC2 in the endosperm possibly suppress mitotic cycle progress of the synergid nucleus; and 4) the ensuing endosperm mitosis induces mitotic catastrophe resulting in the selective nuclear elimination of synergid.

This selective nuclei elimination also exists and has been well studied in animals. The syncytiotrophoblast (STB) in the mammalian placenta is a layer of epithelium that acts as a filter separating the fetal and maternal circulatory systems but allowing exchanges of gas, nutrition, and waste [4, 5]. The STB is multinucleated and the nuclei are recruited by continuous cell-fusions with cytotrophoblasts (CTB). CTBs play key roles in growth and homeostasis in the STB by supplying fresh cellular components through cell fusions. Interestingly, the STB makes characteristic nuclear clusters such as syncytial sprouts and syncytial knots, where old or damaged nuclei are thought to be collected and extruded for maternal circulation. Indeed, nuclei in the syncytial knot exhibit heterochromatin features characteristic of apoptosis. A subset of the nuclei displays low levels of Bcl-2 and Mcl-1 apoptosis inhibitory proteins, suggesting a well-controlled turnover system mediated by programmed-cell-death in the SBT [5]. Nuclear sorting through the formation of a special domain for nuclear deportation appears to be key for selective nuclear elimination in the STB.

As shown above, the mechanisms of nuclear elimination are quite different in the mammalian STB and plant endosperm, despite their functional and structural similarities. Unsynchronized mitotic catastrophe has also been reported in mammalian syncytium produced by artificial fusions of two cells in different mitotic stages [6]. However, nuclei from the CTB cease to proliferate after their incorporation into the STB [4]; therefore, this type of nuclear elimination is unlikely to occur in the STB. So what is the take home message? The mechanistic differences in nuclear elimination may be due to their divergent primary purposes: homeostasis and rapid inactivation of a target cell. Ancestral organisms would have evolved unique mechanisms for selective nuclear elimination. Further molecular dissections and comparisons of animal and plant nuclear elimination mechanisms should reveal the secret of fate-determination and recognition of the target nucleus, the foundation of selective nuclear elimination.
